# Supplementary material for: Opinions towards Medical Students’ Self-Care and Substance Use Dilemmas—A Future Concern despite a Positive Generational Effect?
Source: Int J Environ Res Public Health. 2022 Oct 14;19(20):13289. doi: 10.3390/ijerph192013289 (PMC9603267; doi:10.3390/ijerph192013289)
Supplement: Supplementary file 1 [file ijerph-19-13289-s001.zip › ijerph-1891526-supplementary/S1. Doctor survey.pdf]

Doctors' Survey on Medical Student Professional Dilemmas

Introduction

Dear Colleague,

Thank you for your interest in this project. This survey should take 10 minutes to complete.
The survey aims to:
1. determine how you view medical student professionalism
2. identify what areas of professionalism teaching may require further development
3. examine the role of context in professionalism

This research project has ethics approval by UWA HREC. Your participation is voluntary and your anonymity will be ensured.
The return of completed or part-completed questionnaires will be accepted as an indication that you have consented to participate in the research.
You may withdraw from the survey at any time. Your responses will be anonymous and will not be used individually. This means that it will also not be possible to remove your responses from the database set collected should you wish to withdraw them later.

The scenarios in the survey are designed to encourage reflection; not all of the responses are 'unacceptable'.
If you have any questions or concerns you are welcome to contact the research team (email details below).

Thanks for participating in this study which should provide valuable information into this important area of medical education.

Dr Kiran Narula
A/Prof Christine Jorm
Dr Katrina Calvert
Dr Paul McGurgan (paul.mcgurgan@uwa.edu.au)

As we are interested in finding out if different groups of people have different views on medical student professionalism, we would like to find out some general information about you e.g. gender/age etc:

Are you male or female?

- ○ Male
- ○ Female
- ○ Prefer not to answer

What age category are you?

- ○ Under 25
- ○ 25-35
- ○ 36-45
- ○ 46-55
- ○ 56-65
- ○ 66-75
- ○ Over 75

What is your current grade of practice?

- Intern
- RMO
- Non-training registrar
- Training registrar
- Qualified specialist- consultant
- Qualified specialist- GP

Other (please specify)

What area of practice do you work the *majority* of your time as a doctor?

- N/A- retired
- General practice
- Anaesthesia/Pain medicine/ICU
- Emergency medicine
- Surgery- incl subspecialties eg. max/fax, ENT, orthopaedics, cardiothoracic, neuro, urology, vascular, paeds, plastics, opthalmology
- Medical Administration
- Medicine incl subspecialties eg. paeds, renal, dermatology, endocrine, palliative, sexual health, rehab, occ, clinical genetics, infectious diseases, addiction, oncology, sports medicine
- Obstetrics and gynaecology incl subspecialties
- Radiology incl subspecialties eg. ultrasound, nuclear medicine, radiation oncology
- Pathology incl subspecialties
- Psychiatry incl subspecialties
- Pharmaceuticals/Industry
- Academia- clinical research
- Academia- medical education

Are you involved in teaching/ working with medical students?

- Yes
- No

What percentage of time in your working life do you spend teaching and/or working with medical students?

- Less than 5%
- 5-50%
- 51-95%
- More than 95%

If you wish you can provide more information here:

Doctors' Survey on Medical Student Professional Dilemmas

Main survey: There are 20 professional dilemmas to complete

The scenarios in the survey are designed to cover challenging areas in medical students work and personal lives. Not all of the responses are 'Unacceptable'.

Q 1. A senior medical student is performing their obstetric clerkship. They conduct routine antenatal examinations in the antenatal clinic under the supervision of a consultant. Despite not having experienced problems measuring blood pressure (BP) on patients before, today the senior student is unable to hear any discernible Korotkoff sounds. As this is a fundamental skill, the student decides to fabricate the result and say the BPs are all around 125/70. The fabrication is detected because the consultant is surprised when one of the antenates they knew had essential hypertension has a 'normal' pregnancy BP recorded. The consultant re-checks the patient's blood pressure and discovers that the stethoscope is not working.

This student's behaviour is:

Q 1. A 2nd year medical student is performing their obstetric clerkship. They conduct routine antenatal examinations in the antenatal clinic under the supervision of a consultant. Despite not having experienced problems measuring blood pressure (BP) on patients before, today the 2nd year student is unable to hear any discernible Korotkoff sounds. As this is a fundamental skill, the student decides to fabricate the result and say the BPs are all around 125/70. The fabrication is detected because the consultant is surprised when one of the antenates they knew had essential hypertension has a 'normal' pregnancy BP recorded. The consultant re-checks the patient's blood pressure and discovers that the stethoscope is not working.

This student's behaviour is:

- ○ Acceptable
- ○ Mostly Acceptable
- ○ Mostly Unacceptable
- ○ Unacceptable

You are welcome to comment on this:

Q 2. During a surgical rotation, students' attendance at morning tutorials is compulsory. This is monitored by requiring students to sign in for each tutorial. Student A over-sleeps and texts their friend (Student B) to sign in for them. The senior registrar notes the discrepancy between the number of students in attendance and the number of signatures. When asked to explain, the student who forged the signature (Student B) admits this immediately.

Student A's behaviour is:

Q2. During a surgical rotation, students' attendance at morning tutorials is compulsory. This is monitored by requiring students to sign in for each tutorial. Student A over-sleeps and texts their friend (Student B) to sign in for them. The senior registrar notes the discrepancy between the number of students in attendance and the number of signatures. When asked to explain, the student who forged the signature (Student B) admits this immediately.

Student B's behaviour is:

- ◯ Acceptable
- ◯ Mostly Acceptable
- ◯ Mostly Unacceptable
- ◯ Unacceptable

You are welcome to comment on this:

Q 3. A medical student on their surgery term takes scrubs from the department to use as overalls when repainting a room in their house.

This student's behaviour is:

Q 3. A medical student on their surgery term takes suturing equipment from the department in order to practice this skill at home.

This student's behaviour is:

- ◯ Acceptable
- ◯ Mostly Acceptable
- ◯ Mostly Unacceptable
- ◯ Unacceptable

You are welcome to comment on this:

A 50.0%

Q 4. A medical student has symptoms suggestive of viral gastroenteritis (diarrhoea and vomiting) in the last 24 hours. They have a full day of shadowing on the hospital wards and decide they will not attend.

This student's behaviour is:

B 50.0%

Q 4. A medical student has symptoms suggestive of viral gastroenteritis (diarrhoea and vomiting) in the last 24 hours. They have a full day of shadowing on the hospital wards and decide they will attend.

This student's behaviour is:

- Acceptable
- Mostly Acceptable
- Mostly Unacceptable
- Unacceptable

You are welcome to comment on this:

A 50.0%

Q 5. A final year medical student is attempting to practice their clinical examination skills in preparation for their practical summative assessment. Despite being polite and respectful, the student's request to perform examinations is repeatedly declined by various patients. Stressed by looming exams, the student states they are a doctor in order to examine a patient. This is overheard by a ward nurse and the student is reported to their clinical supervisor.

This student's behaviour is:

B 50.0%

Q 5. A medical student on an emergency department team is assisting in the management of a patient. The team requires a copy of the patient's recent blood results from the patient's GP. At the team's instruction, the medical student is told to call the GP and state they are a doctor from the hospital in order to have the GP fax across this information.

This student's behaviour is:

- Acceptable
- Mostly Acceptable
- Mostly Unacceptable
- Unacceptable

You are welcome to comment on this:

A 50.0%

Q 6. A 4th year student is completing their GP placement. The student notes that the GP does not use gloves whilst performing minor operations.

The GP later asks the student to perform a skin resection for a small lesion on a patient's arm. The student prepares their surgical equipment. When they ask for gloves, the GP replies, "Gloves are unnecessary, this is an 80 year old woman with no risk factors for blood borne viruses and a probable basal cell lesion- go ahead".

The student performs the procedure without gloves.

This student's behaviour is:

B 50.0%

Q 6. A 4th year student is completing their GP placement. The student notes that the GP does not use gloves whilst performing minor operations.

The GP later asks the student to perform a skin resection for a small lesion on a patient's arm. The student prepares their surgical equipment. When they ask for gloves, the GP replies, "Gloves are unnecessary, this is an 80 year old woman with no risk factors for blood borne viruses and a probable basal cell lesion- go ahead".

The student says that they do not wish to proceed without gloves.

This student's behaviour is:

- Acceptable
- Mostly Acceptable
- Mostly Unacceptable
- Unacceptable

You are welcome to comment on this:

Doctors' Survey on Medical Student Professional Dilemmas

[illegible]

Q 7. As part of completing a patient assignment, a student requires patient information from the hospital notes. As the patient notes are not allowed to leave the ward, the student uses their mobile phone to take photographs of the notes to use when writing up their assignment.

This student's behaviour is:

B 50.0%

Q 7. A student on a medical elective in Africa posts photos of the hospital in which they are working on social media. Some of these photos include the faces of patients under their care. The student provides a link to a fund raising organisation on the same page.

This student's behaviour is:

- Acceptable
- Mostly Acceptable
- Mostly Unacceptable
- Unacceptable

You are welcome to comment on this:

[illegible]

Q 8. A female medical student bumps into a 25 year old man at an evening concert. The student had taken his history and performed an abdominal examination in the Emergency Department a fortnight ago when he had attended with abdominal pain. The pair get chatting and the man invites the student back to his flat for "somewhere quieter for a drink". The female student accepts the invite.

This student's behaviour is:

B 50.0%

Q 8. A male medical student bumps into a 25 year old woman at an evening concert. The student had taken her history and performed an abdominal examination in the Emergency Department a fortnight ago when she had attended with abdominal pain. The pair get chatting and the woman invites the student back to her flat for "somewhere quieter for a drink". The male student accepts the invite.

This student's behaviour is:

- Acceptable
- Mostly Acceptable
- Mostly Unacceptable
- Unacceptable

You are welcome to comment on this:

[illegible] Q 9. A second year medical student posts a comment on Facebook stating 'This country cannot afford to throw away $$$ treating fat people who do not take care of their own health'.

This student's behaviour is:

[illegible]

Q 9. A second year medical student posts a comment on Facebook stating 'This country cannot afford to throw away $$$ treating Indigenous people who do not take care of their own health'.

This student's behaviour is:

- Acceptable
- Mostly Acceptable
- Mostly Unacceptable
- Unacceptable

You are welcome to comment on this:

[illegible]

Q 10. A 36 year old insulin dependent diabetic is admitted via the Emergency Department to the Medical Assessment Unit (MAU) with sepsis and poor glucose control. The patient is married to a female final year medical student who is very concerned about him. The MAU staff are readily available to answer any queries, but the medical student decides to log in to the hospital computer system to check her husband's pathology lab results.

This student's behaviour is:

[illegible]

Q 10. A 36 year old insulin dependent diabetic is admitted via the Emergency Department to the Medical Assessment Unit (MAU) with sepsis and poor glucose control. The patient is married to a female final year medical student who is very concerned about him. The MAU staff appear to be very busy so the medical student decides to log in to the hospital computer system to check her husband's pathology lab results.

This student's behaviour is:

- ○ Acceptable
- ○ Mostly Acceptable
- ○ Mostly Unacceptable
- ○ Unacceptable

You are welcome to comment on this:

[illegible]

Q 11. The Medical School is informed that a 4th year medical student (postgraduate entry 4 year course) has been charged with drunk and disorderly conduct after an altercation at a night club.

This student's behaviour is:

[illegible]

Q 11. The Medical School is informed that a 1st year medical student (undergraduate entry 6 year course) has been charged with drunk and disorderly conduct after an altercation at a night club.

This student's behaviour is:

- ○ Acceptable
- ○ Mostly Acceptable
- ○ Mostly Unacceptable
- ○ Unacceptable

You are welcome to comment on this:

Q 12. A medical student rushes to an emergency bell on the ward. They are the first responder and commence effective CPR. Despite the arrival and assistance of the Medical Emergency Team the patient dies.
The student is distressed by recurring thoughts of the event, which affect their sleep. When these symptoms continue, the student seeks assistance from Student Support Services.

This student's behaviour is:

- ◯ Acceptable
- ◯ Mostly acceptable
- ◯ Mostly unacceptable
- ◯ Unacceptable

You are welcome to comment on this:

A 50.0%

Q 13. During exam time a first year medical student buys stimulant drugs online that are usually only available on prescription, and uses them in order to stay awake and study.

This student's behaviour is:

B 50.0%

Q 13. During exam time a final year medical student buys stimulant drugs online that are usually only available on prescription, and uses them in order to stay awake and study.

This student's behaviour is:

- Acceptable
- Mostly Acceptable
- Mostly Unacceptable
- Unacceptable

You are welcome to comment on this:

A 50.0%

Q 14. A final year medical student in an undergraduate entry medical course fails all of their end of year examinations at the first attempt. The student had not applied for any special consideration, but subsequently admits that their ability to study had been affected by anxiety and they had used cannabis daily to provide relief over the past 2 months.

This student's behaviour is:

B 50.0%

Q 14. A first year medical student in an undergraduate entry medical course fails all of their end of year examinations at the first attempt. The student had not applied for any special consideration, but subsequently admits that their ability to study had been affected by anxiety and they had used cannabis daily to provide relief over the past 2 months.

This student's behaviour is:

- Acceptable
- Mostly Acceptable
- Mostly Unacceptable
- Unacceptable

You are welcome to comment on this:

A 50.0% Q 15. A member of the public complains to the medical school that one of their male students works as part of a male stripping troupe. The student is in 4th year. When he is identified he states "I need the money to survive, what I do in my own time if legal is my own business".

This student's behaviour is:

B 50.0%

Q 15. A member of the public complains to the medical school that one of their female students works as a topless lap dancer in a bar. The student is in 4th year. When she is identified she states "I need the money to survive, what I do in my own time if legal is my own business".

This student's behaviour is:

- Acceptable
- Mostly Acceptable
- Mostly Unacceptable
- Unacceptable

You are welcome to comment on this:

A 50.0% Q 16. During a paediatric rotation, a student doctor is rostered onto the same shift as his girlfriend, who is a midwife on the neonatal ward. The student accompanies the midwife on a round, and witnesses her accidentally administer a 2 day old baby with an adult dose of Hep B vaccine. The student is aware that the high dose is unlikely to have any serious ill effects, and decides not to say anything as he does not wish his girlfriend to get into trouble.

The student's behaviour is:

B 50.0% Q 16. During a paediatric rotation, a student doctor is rostered onto the same shift as his girlfriend, who is a junior doctor on the neonatal ward. The student accompanies the doctor on a round, and witnesses her accidentally administer a 2 day old baby with an adult dose of Hep B vaccine. The student is aware that the high dose is unlikely to have any serious ill effects, and decides not to say anything as he does not wish his girlfriend to get into trouble.

The student's behaviour is:

- Acceptable
- Mostly acceptable
- Mostly unacceptable
- Unacceptable

You are welcome to comment on this:

Doctors' Survey on Medical Student Professional Dilemmas

A 50.0% Q 17. A hospital education program includes mandatory attendance multidisciplinary teaching for student doctors, nurses, and allied health professionals. A mandatory teaching session run by the hospital's senior dietician has been scheduled for Monday morning at 0800. A third year medical student with exams approaching decides that their time would be better spent studying, and does not attend the session.

The student's behaviour is:

B 50.0% Q 17. A hospital education program includes mandatory attendance multidisciplinary teaching for student doctors, nurses, and allied health professionals. A mandatory teaching session run by the hospital's senior cardiologist has been scheduled for Monday morning at 0800. A third year medical student with exams approaching decides that their time would be better spent studying, and does not attend the session.

The student's behaviour is:

- ◯ Acceptable
- ◯ Mostly acceptable
- ◯ Mostly unacceptable
- ◯ Unacceptable

You are welcome to comment on this:

A 50.0% Q 18. A junior doctor on a surgical team advises her medical student friend that the senior doctor on the team "thinks he's a bit of a lad", and that all she needs to do to get a good reference is lower her neckline and flirt a little. The following week, the medical student wears something more revealing and says to the consultant "I cannot believe how you got to be a consultant with your level of experience when you look so young".

The student's behaviour is:

B 50.0% Q 18. A junior doctor on a surgical team advises his medical student friend that the senior doctor on the team "thinks she's a bit of a cougar", and that all he needs to do to get a good reference is wear a tight fitting shirt and flirt a little. The following week, the medical student wears something more revealing and says to the consultant "I cannot believe how you got to be a consultant with your level of experience when you look so young".

The student's behaviour is:

- ◯ Acceptable
- ◯ Mostly acceptable
- ◯ Mostly unacceptable
- ◯ Unacceptable

You are welcome to comment on this:

A 50.0% Q 19. A final year student is on an obstetrics rotation and is required to complete two labour ward night shifts. The student is a single parent with difficulties arranging childcare at home, and misses the last night shift. As completion of two night shifts is a compulsory part of the term, the student forges a signature from the senior midwife confirming that the shifts were completed.

The student's behaviour is:

B 50.0% Q 19. A final year student is on an obstetrics rotation and is required to complete two labour ward night shifts. The student is invited to a friend's engagement party, and misses the last night shift. As completion of two night shifts is a compulsory part of the term, the student forges a signature from the senior midwife confirming that the shifts were completed.

The student's behaviour is:

- Acceptable
- Mostly acceptable
- Mostly unacceptable
- Unacceptable

You are welcome to comment on this:

A 50.0% Q 20. A medical student is sitting in on a practice nurse clinic in a general practice. The nurse is discussing vaccinations with parents of young children. The student notices that the nurse is providing much more information to white Australian parents than to parents of other ethnicities, irrespective of their language skills. After the clinic the student asks the nurse about this. The nurse replies, "Oh, it doesn't matter, those sorts of people always do what we tell them to, so we don't need to worry too much about the counselling, we can just tell them to do it". The student considers this behaviour to be racist, but does not report the nurse as the student does not wish to get them into trouble.

The student's behaviour is:

B 50.0% Q 20. A medical student is sitting in on a clinic in a general practice. The GP trainee registrar is discussing vaccinations with parents of young children. The student notices that the GP trainee is providing much more information to white Australian parents than to parents of other ethnicities, irrespective of their language skills. After the clinic the student asks the GP trainee about this. The GP trainee replies, "Oh, it doesn't matter, those sorts of people always do what we tell them to, so we don't need to worry too much about the counselling, we can just tell them to do it". The student considers this behaviour to be racist, but does not report the GP trainee as the student does not wish to get them into trouble.

The student's behaviour is:

- Acceptable
- Mostly acceptable
- Mostly unacceptable
- Unacceptable

You are welcome to comment on this:

Doctors' Survey on Medical Student Professional Dilemmas

Conclusion

How do you rate the overall professionalism of medical students at present?

- Similar to when I was a student
- Better than when I was a student
- Worse than when I was a student

If you wish you can provide more information here:

Were you an undergraduate or post-graduate entry to your medical school?

- Undergraduate entry
- Postgraduate entry

Did you complete your medical degree in Australia or elsewhere?

- Australia
- New Zealand
- UK/Ireland
- Europe/Russia
- Indian subcontinent
- Asia
- Africa
- North America/Canada
- South/Central America

Did you have any professionalism breaches or formal complaints made against you when you were a medical student?

- Yes
- No

You are welcome to provide more information below:

You are welcome to add any comments you have on this survey or the issue of medical student or doctor's professionalism in the box below:

Thank you for your participation in this survey.
If you have any queries or concerns please contact the researchers:

Dr. Paul McGurgan,
c/o School of Women's and Infants' Health,
The University of Western Australia (M550),
35 Stirling Highway,
CRAWLEY WA 6009, Australia.

Phone: +61 8 9340 1330
e mail: paul.mcgurgan@uwa.edu.au
