# Supplementary material for: Opinions towards Medical Students’ Self-Care and Substance Use Dilemmas—A Future Concern despite a Positive Generational Effect?
Source: Int J Environ Res Public Health. 2022 Oct 14;19(20):13289. doi: 10.3390/ijerph192013289 (PMC9603267; doi:10.3390/ijerph192013289)
Supplement: Supplementary file 1 [file ijerph-19-13289-s001.zip › ijerph-1891526-supplementary/S2. Public survey.pdf]

Australian Public's Survey on Medical Student Professionalism Dilemmas

Introduction

Thank you for your interest in this project. The survey should take 10 minutes to complete.

Being a professional can mean different things to different people. Medical students are university students training to be doctors. Whereas qualified doctors have strict rules on professional behaviours, medical students have less clear guidance as they are not yet working as doctors.

The survey aims to:
1. determine how members of the public view medical student professionalism
2. examine whether different groups of people view professionalism differently

The project has ethics approval by University of Western Australia. Your participation is voluntary and your anonymity will be ensured.
The return of completed or part-completed questionnaires will be accepted as an indication that you have consented to participate in the research.
You may withdraw from the survey at any time. Your responses will be anonymous and will not be used individually. This means that it will also not be possible to remove your responses from the database set collected should you wish to withdraw them later.

The scenarios in the survey are designed to cover challenging areas in medical students' work and personal lives. Not all of the responses are 'Unacceptable'.
If you have any questions or concerns you are welcome to contact the research team (email details below).

Thanks for participating in this study which should provide valuable information into this important area of medical education.

Mr Kiran Narula
A/Prof Christine Jorm
Dr Katrina Calvert
Dr Paul McGurgan (paul.mcgurgan@uwa.edu.au)

As we are interested in finding out if different groups of people have different views on medical student professionalism, we would like to find out some general information about you (gender/age etc.):

What age category are you?

- ○ Under 25
- ○ 25-35
- ○ 36-45
- ○ 46-55
- ○ 56-65
- ○ 66-75
- ○ Over 75

Are you male or female?

- ○ Male
- ○ Female
- ○ Prefer not to answer

Were you born in Australia or overseas?

- ○ Born in Australia
- ○ Born overseas

What is the highest level of education you completed?

- ◯ Up to Year 12 or left school before 18 years old
- ◯ Tertiary e.g. TAFE or University certificate/diploma/degree
- ◯ Post-graduate qualification e.g. Masters/PhD

Have you worked in health care with medical students or doctors in any capacity?

- ◯ Yes
- ◯ No

If you wish, you can provide more information here:

Have you or someone close to you had a bad health care experience involving Australian doctors or medical students?

- ◯ Yes
- ◯ No

If you wish, you can provide more information here:

Australian Public's Survey on Medical Student Professionalism Dilemmas

Main survey: There are 20 professional dilemmas to complete

Q 1. A senior medical student is working with a doctor in clinic. The student has been taught how to measure patients' blood pressures.
Despite not having experienced problems measuring blood pressure (BP) on patients before, today the senior student is unable to obtain the measurement. As this is a basic skill, the student decides to make up the result and say the BPs are all around 125/70 (normal).
The falsehood is detected when the doctor re-checks the patient's blood pressure and discovers that the machine is not working.

This student's behaviour is:

Q 1. A junior medical student is working with a doctor in clinic. The student has been taught how to measure patients' blood pressures.
Despite not having experienced problems measuring blood pressure (BP) on patients before, today the junior student is unable to obtain the measurement. As this is a basic skill, the student decides to make up the result and say the BPs are all around 125/70 (normal).
The falsehood is detected when the doctor re-checks the patient's blood pressure and discovers that the machine is not working.

This student's behaviour is:

- Acceptable
- Mostly Acceptable
- Mostly Unacceptable
- Unacceptable

You are welcome to comment on this:

A 50.0%

Q 2. When studying at a hospital, medical students' attendance at morning tutorials is compulsory. This is monitored by requiring students to sign in for each tutorial.
Student A over-sleeps and texts their friend (Student B) to sign in for them. The senior doctor notes the difference between the number of students in attendance and the number of signatures.
When asked to explain, the student who forged the signature (Student B) admits this immediately.

Student A's behaviour is:

B 50.0%

Q2. When studying at a hospital, medical students' attendance at morning tutorials is compulsory. This is monitored by requiring students to sign in for each tutorial.
Student A over-sleeps and texts their friend (Student B) to sign in for them. The senior doctor notes the difference between the number of students in attendance and the number of signatures.
When asked to explain, the student who forged the signature (Student B) admits this immediately.

Student B's behaviour is:

- Acceptable
- Mostly Acceptable
- Mostly Unacceptable
- Unacceptable

You are welcome to comment on this:

A 50.0%

Q 3. A medical student on their surgery term takes 'scrubs' (the clothes used by doctors in theatre) from the hospital to use as overalls when repainting a room in their house.

This student's behaviour is:

B 50.0%

Q 3. A medical student on their surgery term takes suturing equipment from the hospital in order to practice this skill at home.

This student's behaviour is:

- Acceptable
- Mostly Acceptable
- Mostly Unacceptable
- Unacceptable

You are welcome to comment on this:

A 50.0%

Q 5. A final year medical student is attempting to practice their clinical examination skills in preparation for their final exams. Despite being polite and respectful, the student's request to perform examinations is repeatedly declined by various patients.
Stressed by looming exams, the student states they are a doctor in order to examine a patient. This is overheard by a ward nurse and the student is reported to their clinical supervisor.

This student's behaviour is:

B 50.0%

Q 5. A medical student is assisting in the management of a patient in the Emergency Department. The medical team requires a copy of the patient's recent blood results from the patient's General Practitioner (GP). At the team's instruction, the medical student is told to call the GP and state they are a doctor from the hospital in order to have the GP fax across this information.

This student's behaviour is:

- Acceptable
- Mostly Acceptable
- Mostly Unacceptable
- Unacceptable

You are welcome to comment on this:

[illegible] Q 4. A medical student has symptoms suggestive of a viral illness (diarrhoea and vomiting) in the last 24 hours. They have a full day of working on the hospital wards and decide they will not attend.

This student's behaviour is:

[illegible]

Q 4. A medical student has symptoms suggestive of a viral illness (diarrhoea and vomiting) in the last 24 hours. They have a full day of working on the hospital wards and decide they will attend.

This student's behaviour is:

- ◯ Acceptable
- ◯ Mostly Acceptable
- ◯ Mostly Unacceptable
- ◯ Unacceptable

You are welcome to comment on this:

[illegible]

Q 6. A medical student is completing their General Practice (GP) placement. The student notes that the GP does not use gloves whilst performing minor operations.

The GP later asks the student to perform a minor skin operation for a small lesion on a patient's arm. The student prepares their surgical equipment. When they ask for gloves, the GP replies, "Gloves are unnecessary, this is a minor skin problem- go ahead".

The student performs the procedure without gloves.

This student's behaviour is:

[illegible]

Q 6. A medical student is completing their General Practice (GP) placement. The student notes that the GP does not use gloves whilst performing minor operations.

The GP later asks the student to perform a minor skin operation for a small lesion on a patient's arm. The student prepares their surgical equipment. When they ask for gloves, the GP replies, "Gloves are unnecessary, this is a minor skin problem - go ahead".

The student says that they do not wish to proceed without gloves.

This student's behaviour is:

- Acceptable
- Mostly Acceptable
- Mostly Unacceptable
- Unacceptable

You are welcome to comment on this:

A 50.0%

Q 7. As part of completing a patient assignment, a medical student requires patient information from the hospital notes. As the patient notes are not allowed to leave the ward, the student uses their mobile phone to take photographs of the notes to use when writing up their assignment.

This student's behaviour is:

B 50.0%

Q 7. A medical student on elective in Africa posts photos of the hospital in which they are working on social media. Some of these photos include the faces of patients under their care. The student provides a link to a fund raising organisation on the same page.

This student's behaviour is:

- Acceptable
- Mostly Acceptable
- Mostly Unacceptable
- Unacceptable

You are welcome to comment on this:

A 50.0%

Q 8. A female medical student bumps into a 25 year old man at an evening concert. The student had taken his history and performed an abdominal examination in the Emergency Department a fortnight ago when he had attended with abdominal pain. The pair get chatting and the man invites the student back to his flat for “somewhere quieter for a drink”. The female student accepts the invite.

This student's behaviour is:

B 50.0%

Q 8. A male medical student bumps into a 25 year old woman at an evening concert. The student had taken her history and performed an abdominal examination in the Emergency Department a fortnight ago when she had attended with abdominal pain. The pair get chatting and the woman invites the student back to her flat for “somewhere quieter for a drink”. The male student accepts the invite.

This student's behaviour is:

- Acceptable
- Mostly Acceptable
- Mostly Unacceptable
- Unacceptable

You are welcome to comment on this:

[illegible]

Q 9. A second year medical student posts a comment on Facebook stating 'This country cannot afford to throw away $$$ treating fat people who do not take care of their own health'.

This student's behaviour is:

[illegible]

Q 9. A second year medical student posts a comment on Facebook stating 'This country cannot afford to throw away $$$ treating Indigenous people who do not take care of their own health'.

This student's behaviour is:

- Acceptable
- Mostly Acceptable
- Mostly Unacceptable
- Unacceptable

You are welcome to comment on this:

[illegible]

Q 10. A 36 year old insulin dependent diabetic is admitted to hospital with infection and poor sugar control. The patient is married to a female final year medical student who is very concerned about him. The staff are readily available to answer any queries, but the medical student decides to log in to the hospital computer system to check her husband's blood test results.

This student's behaviour is:

[illegible]

Q 10. A 36 year old insulin dependent diabetic is admitted to hospital with infection and poor sugar control. The patient is married to a female final year medical student who is very concerned about him. The staff appear to be very busy so the medical student decides to log in to the hospital computer system to check her husband's blood test results.

This student's behaviour is:

- Acceptable
- Mostly Acceptable
- Mostly Unacceptable
- Unacceptable

You are welcome to comment on this:

A 50.0%

Q 11. The Medical School is informed that a final year medical student has been charged with drunk and disorderly conduct after an altercation at a night club.

This student's behaviour is:

B 50.0%

Q 11. The Medical School is informed that a 1st year medical student has been charged with drunk and disorderly conduct after an altercation at a night club.

This student's behaviour is:

- Acceptable
- Mostly Acceptable
- Mostly Unacceptable
- Unacceptable

You are welcome to comment on this:

Q 12. A medical student rushes to an emergency bell on the ward. They are the first responder and commence effective First Aid. Despite the arrival and assistance of the Medical Emergency Team the patient dies.
The student is distressed by recurring thoughts of the event, which affect their sleep. When these symptoms continue, the student seeks assistance from Student Support Services.

This student's behaviour is:

- Acceptable
- Mostly acceptable
- Mostly unacceptable
- Unacceptable

You are welcome to comment on this:

Q 13. During exam time a first year medical student buys stimulant drugs online that are usually only available on prescription, and uses them in order to stay awake and study.

This student's behaviour is:

Q 13. During exam time a final year medical student buys stimulant drugs online that are usually only available on prescription, and uses them in order to stay awake and study.

This student's behaviour is:

- Acceptable
- Mostly Acceptable
- Mostly Unacceptable
- Unacceptable

You are welcome to comment on this:

A 50.0%

Q 14. A final year medical student fails all of their end of year examinations at the first attempt. The student had not told the teaching staff that they were having problems or sought assistance, but subsequently admits that their ability to study had been affected by anxiety and they had used cannabis daily to provide relief over the past 2 months.

This student's behaviour is:

B 50.0%

Q 14. A first year medical student fails all of their end of year examinations at the first attempt. The student had not told the teaching staff that they were having problems or sought assistance, but subsequently admits that their ability to study had been affected by anxiety and they had used cannabis daily to provide relief over the past 2 months.

This student's behaviour is:

- Acceptable
- Mostly Acceptable
- Mostly Unacceptable
- Unacceptable

You are welcome to comment on this:

A 50.0%

Q 15. A member of the public complains to the medical school that one of their senior male students works as part of a male stripping troupe. When he is identified he states "I need the money to survive, what I do in my own time if legal is my own business".

This student's behaviour is:

B 50.0%

Q 15. A member of the public complains to the medical school that one of their senior female students works as a topless lap dancer in a bar. When she is identified she states "I need the money to survive, what I do in my own time if legal is my own business".

This student's behaviour is:

- Acceptable
- Mostly Acceptable
- Mostly Unacceptable
- Unacceptable

You are welcome to comment on this:

A 50.0%

Q 16. During a hospital rotation, a student doctor is rostered onto the same shift as his girlfriend, who is a midwife on the paediatric ward. The student accompanies the midwife on a round, and witnesses her accidentally administer a 2 day old baby with an adult dose of Hep B vaccine. The student is aware that the high dose is unlikely to have any serious ill effects, and decides not to say anything as he does not wish his girlfriend to get into trouble.

The student's behaviour is:

B 50.0%

Q 16. During a hospital rotation, a student doctor is rostered onto the same shift as his girlfriend, who is a junior doctor on the paediatric ward. The student accompanies the doctor on a round, and witnesses her accidentally administer a 2 day old baby with an adult dose of Hep B vaccine. The student is aware that the high dose is unlikely to have any serious ill effects, and decides not to say anything as he does not wish his girlfriend to get into trouble.

The student's behaviour is:

- Acceptable
- Mostly acceptable
- Mostly unacceptable
- Unacceptable

You are welcome to comment on this:

Q 17. A hospital education program includes compulsory attendance for student doctors, nurses, and allied health professionals. A compulsory teaching session run by the hospital's senior dietician has been scheduled for Monday morning at 08.00. A third year medical student with exams approaching decides that their time would be better spent studying, and does not attend the session.

The student's behaviour is:

Q 17. A hospital education program includes compulsory attendance for student doctors, nurses, and allied health professionals. A compulsory teaching session run by the hospital's senior cardiologist has been scheduled for Monday morning at 08.00. A third year medical student with exams approaching decides that their time would be better spent studying, and does not attend the session.

The student's behaviour is:

- Acceptable
- Mostly acceptable
- Mostly unacceptable
- Unacceptable

You are welcome to comment on this:

Q 18. A junior doctor on a surgical team advises her medical student friend that the senior doctor on the team "thinks he's a bit of a lad", and that all she needs to do to get a good reference is lower her neckline and flirt a little. The following week, the medical student wears something more revealing and says to the consultant "I cannot believe how you got to be a consultant with your level of experience when you look so young".

The student's behaviour is:

Q 18. A junior doctor on a surgical team advises his medical student friend that the senior doctor on the team "thinks she's a bit of a cougar", and that all he needs to do to get a good reference is wear a tight fitting shirt and flirt a little. The following week, the medical student wears something more revealing and says to the consultant "I cannot believe how you got to be a consultant with your level of experience when you look so young".

The student's behaviour is:

- Acceptable
- Mostly acceptable
- Mostly unacceptable
- Unacceptable

You are welcome to comment on this:

Australian Public's Survey on Medical Student Professionalism Dilemmas

A 50.0% Q 19. A final year student is on a hospital rotation is required to complete two labour ward night shifts. The student is a single parent with difficulties arranging childcare at home, and misses the last night shift. As completion of two night shifts is a compulsory part of the term, the student forges a signature from the senior midwife confirming that the shifts were completed.

The student's behaviour is:

B 50.0% Q 19. A final year student is on a hospital rotation and is required to complete two labour ward night shifts. The student is invited to a friend's engagement party, and misses the last night shift. As completion of two night shifts is a compulsory part of the term, the student forges a signature from the senior midwife confirming that the shifts were completed.

The student's behaviour is:

- Acceptable
- Mostly acceptable
- Mostly unacceptable
- Unacceptable

You are welcome to comment on this:

A 50.0% Q 20. A medical student is sitting in on a practice nurse clinic in a general practice. The nurse is discussing vaccinations with parents of young children. The student notices that the nurse is providing much more information to white Australian parents than to parents of other ethnicities, irrespective of their language skills. After the clinic the student asks the nurse about this. The nurse replies, "Oh, it doesn't matter, those sorts of people always do what we tell them to, so we don't need to worry too much about the counselling, we can just tell them to do it". The student considers this behaviour to be racist, but does not report the nurse as the student does not wish to get them into trouble.

The student's behaviour is:

B 50.0% Q 20. A medical student is sitting in on a clinic in a general practice. The GP trainee registrar is discussing vaccinations with parents of young children. The student notices that the GP trainee is providing much more information to white Australian parents than to parents of other ethnicities, irrespective of their language skills. After the clinic the student asks the GP trainee about this. The GP trainee replies, "Oh, it doesn't matter, those sorts of people always do what we tell them to, so we don't need to worry too much about the counselling, we can just tell them to do it". The student considers this behaviour to be racist, but does not report the GP trainee as the student does not wish to get them into trouble.

The student's behaviour is:

- Acceptable
- Mostly acceptable
- Mostly unacceptable
- Unacceptable

You are welcome to comment on this:

Australian Public's Survey on Medical Student Professionalism Dilemmas

Conclusion

How do you rate the overall professionalism of medical students at present?

- Unable to comment
- Similar to previous years
- Better than previous years
- Worse than previous years

You are welcome to add any comments you have on this survey or the issue of medical student or doctor's professionalism in the box below:

Thank you for your participation in this survey.
If you have any queries or concerns please contact the researchers:

Dr. Paul McGurgan,
c/o School of Women's and Infants' Health,
The University of Western Australia (M550),
35 Stirling Highway,
CRAWLEY WA 6009, Australia.

Phone: +61 8 9340 1330
e mail: paul.mcgurgan@uwa.edu.au
